# Steroidal Lactones from *Withania somnifera*, an Ancient Plant for Novel Medicine

**DOI:** 10.3390/molecules14072373

**Published:** 2009-07-03

**Authors:** Mohammad Hossein Mirjalili, Elisabeth Moyano, Mercedes Bonfill, Rosa M. Cusido, Javier Palazón

**Affiliations:** 1 Medicinal Plants and Drugs Research Institute, Shahid Beheshti University, G.C., Evin, Tehran, Iran; E-mail: m-mirjalili@sbu.ac.ir (M.H.M.); 2 Departament de Ciencies Experimentals i de la Salut, Universitat Pompeu Fabra, Av. Doctor Aiguader 80, 08003 Barcelona, Spain; E-mail: elisabeth.moyano@ub.edu (E.M.); 3 Laboratori de Fisiologia Vegetal, Facultat de Farmacia. Universitat de Barcelona, Av. Joan XXIII s/n, 08028 Barcelona, Spain; E-mails: mbonfill@ub.edu (M.B.), rcusido@ub.edu (R.M.C.)

**Keywords:** *Withania somnifera*, withanolides, withaferin A, withanolide A, triterpenoids

## Abstract

*Withania somnifera*, commonly known as Ashwagandha, is an important medicinal plant that has been used in Ayurvedic and indigenous medicine for over 3,000 years. In view of its varied therapeutic potential, it has also been the subject of considerable modern scientific attention. The major chemical constituents of the *Withania* genus, the withanolides, are a group of naturally occurring C_28_-steroidal lactone triterpenoids built on an intact or rearranged ergostane framework, in which C-22 and C-26 are appropriately oxidized to form a six-membered lactone ring. In recent years, numerous pharmacological investigations have been carried out into the components of *W. somnifera* extracts. We present here an overview of the chemical structures of triterpenoid components and their biological activity, focusing on two novel activities, tumor inhibition and antiangiogenic properties of withaferin A and the effects of withanolide A on Alzheimer's disease. The most recent attempts in biotechnological production of withanolides are also discussed.

## Introduction

The Solanaceae family is comprised of 84 genera that include about 3,000 species, scattered throughout the world. Members of this family are generally annual shrubs. The genera *Withania* and *Physalis* play an important role in the indigenous medicine of South East Asia, e.g. in the Unani and Ayurvedic systems. The twenty-three known *Withania* species are widely distributed in the drier parts of tropical and subtropical zones, ranging from the Canary Islands, the Mediterranean region and northern Africa to Southwest Asia [1,2,3,4]. Among them, only two species, *W. somnifera* and *W. coagulans*, are economically and medicinally significant, being used and cultivated in several regions [5,6,7]. 

*W. somnifera*, commonly known as Ashwagandha, is an important medicinal plant that has been used in Ayurvedic and indigenous medicine for over 3,000 years [8]. In view of its varied therapeutic potential, it has also been the subject of considerable modern scientific attention. Ashwagandha roots are a constituent of over 200 formulations in Ayruvedha, Siddha and Unani medicine, which are used in the treatment of various physiological disorders [9,10]. *Withania* appears in WHO monographs on Selected Medicinal Plants and an American Herbal Pharmacopoeia monograph is also forthcoming [11].

In Ayurveda, *Withania* is widely claimed to have potent aphrodisiac, sedative, rejuvenative and life prolonging properties. It is also used as a general energy-enhancing tonic known as Medharasayana, which means ‘that which promotes learning and a good memory’ and in geriatric problems [12,13]. The plant was traditionally used to promote youthful vigor, endurance, strength, and health, nurturing the time elements of the body and increasing the production of vital fluids, muscle fat, blood, lymph, semen and cells. The similarity between these restorative properties and those of ginseng roots has led to Ashwagandha roots being called Indian ginseng [10]. It also helps counteract chronic fatigue, weakness, dehydration, bone weakness, loose teeth, thirst, impotency, premature ageing, emaciation, debility, and muscle tension. The leaves of the plant are bitter in taste and used as an antihelmantic. The infusion is given in fever. Bruised leaves and fruits are locally applied to tumors and tubercular glands, carbuncles and ulcers [12,14]. The roots are used as a nutrient and health restorative in pregnant women and old people. The decoction of the root boiled with milk and ghee is recommended for curing sterility in women. The roots are also used in constipation, senile debility, rheumatism, general debility, nervous exhaustion, loss of memory, loss of muscular energy and spermatorrhoea [10,15]. Today *W. somnifera* is widely cultivated in the drier parts of India (more than 4,000 ha) i.e. Manasa, Neemuch and Jawad tehsils of the Mandsaur District of Madhya Pradesh, Punjab, Sind and Rajastan [6,8]. 

*W. coagulans*, common in Iran, Pakistan, Afghanistan and East India, is also used in folk medicine. Fruits of the plant have a milk-coagulating property attributed to the pulp and husk of the berry, which has been used in the preparation of vegetable rennet ferment for cheese [16]. In 1884 Sheridan Lea found upon examination that the coagulating substance is a ferment closely resembling animal rennet [17]. The milk-coagulating activity is due to the presence of an enzyme and, under optimum conditions, one part of concentrated enzyme can coagulate 90,000 parts of milk in half an hour [16]. The fruits are reported to be sedative, emetic, and stomachic, a blood-purifier and febrifuge, an alterative, diuretic, and bitter tonic in dyspepsia as well as a growth promoter in infants [15]. They are also useful in chronic complaints of liver, and in the treatment of asthma and biliousness. The twigs are chewed to clean teeth and the smoke of the plant is inhaled to relieve toothache. The leaves are used as a vegetable and as fodder for livestock [12,18]. The crude preparation of the plant has been found to be active against a number of pathogenic bacteria [19].

*W. somnifera* (L.) Dunal (Synon. *Physalis somnifera* L.; *Physalis flexuosa* L.) is an erect, grayish, stellate-tomentose undershrub (30-75 cm high) with long tuberous roots. Leaves are alternate or sub-opposite, broadly ovate to oblong, petiolate, sub-acute, entire, with lamina 5-10 x 2.5-7 cm. Flowers are small, greenish, axillary, solitary or in few-flowered cymes and bisexual. The calyx is gamosepalous with five 3-5 mm lobes, accrescent and inflated in a fruit. The corolla is campanulate, greenish-yellow with five 5-8 mm lobes. There are five included stamens. The ovary is ovoid/globose, glabrous, and many ovuled. The style is filiform and stigma is 2-lobed. Fruit is a globose berry, orange-red when ripe and enclosed in the enlarged calyx. Seeds are many, discoid, yellow and reniform. The chromosome number is 2*n* = 48 [1,2,20].

## Chemical constituents

**Figure 1:**
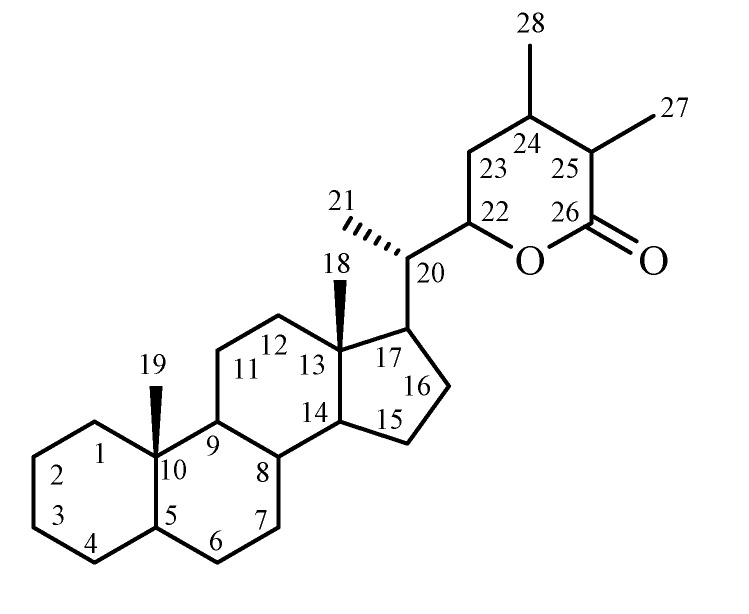
The basic structure of withanolides.

The chemistry of *Withania* species has been extensively studied and several groups of chemical constituents such as steroidal lactones, alkaloids, flavonoids, tannin etc. have been identified, extracted, and isolated [14,21,22,23,24,25]. At present, more than 12 alkaloids, 40 withanolides, and several sitoindosides (a withanolide containing a glucose molecule at carbon 27) have been isolated and reported from aerial parts, roots and berries of *Withania* species. The major chemical constituents of these plants, withanolides, are mainly localized in leaves, and their concentration usually ranges from 0.001 to 0.5% dry weight (DW) [14,26,27]. The withanolides are a group of naturally occurring C_28_-steroidal lactones built on an intact or rearranged ergostane framework, in which C-22 and C-26 are appropriately oxidized to form a six-membered lactone ring. The basic structure (Figure 1) is designated as the withanolide skeleton [28,29,30,31,32]. 

The withanolide skeleton may be defined as a 22-hydroxyergostan-26-oic acid-26,22-lactone. There are many novel structural variants of withanolides with modifications either of the carbocyclic skeleton or the side chain and these have often been described as modified withanolides or ergostan-type steroids related to withanolides. These compounds are generally polyoxygenated and it is believed that plants elaborating them possess an enzyme system capable of oxidizing all carbon atoms in a steroid nucleus. The characteristic feature of withanolides and ergosane-type steroids is one C_8_ or C_9_-side chain with a lactone or lactol ring but the lactone ring may be either six-membered or five-membered and may be fused with the carbocyclic part of the molecule through a carbon-carbon bond or through an oxygen bridge. Appropriate oxygen substituents may lead to bond scission, formation of new bonds, aromatization of rings and many other kinds of rearrangements resulting in compounds with novel structures [28,30,33]. 

Withanolides are present in fifteen Solanaceous genera i.e. *Acnistus*, *Datura*, *Deprea*, *Dunalis*, *Iochroma*, *Jaborosa*, *Lycium*, *Nicandra*, *Physalis*, *Salpichroa*, *Tubocapsicum*, *Discopodium*, *Trechonaetes*, *Withania* and *Witheringia*. The occurrence of withanolides is not completely restricted to Solanaceous plants and reports of their isolation from marine organisms, and from members of the Taccaceae [34,35], Fabaceae (Leguminosae) [36] and Lamiaceae (Labiatae) [37] families suggest that they are much more widely distributed. 

## Biosynthesis of withanolides

**Figure 2:**
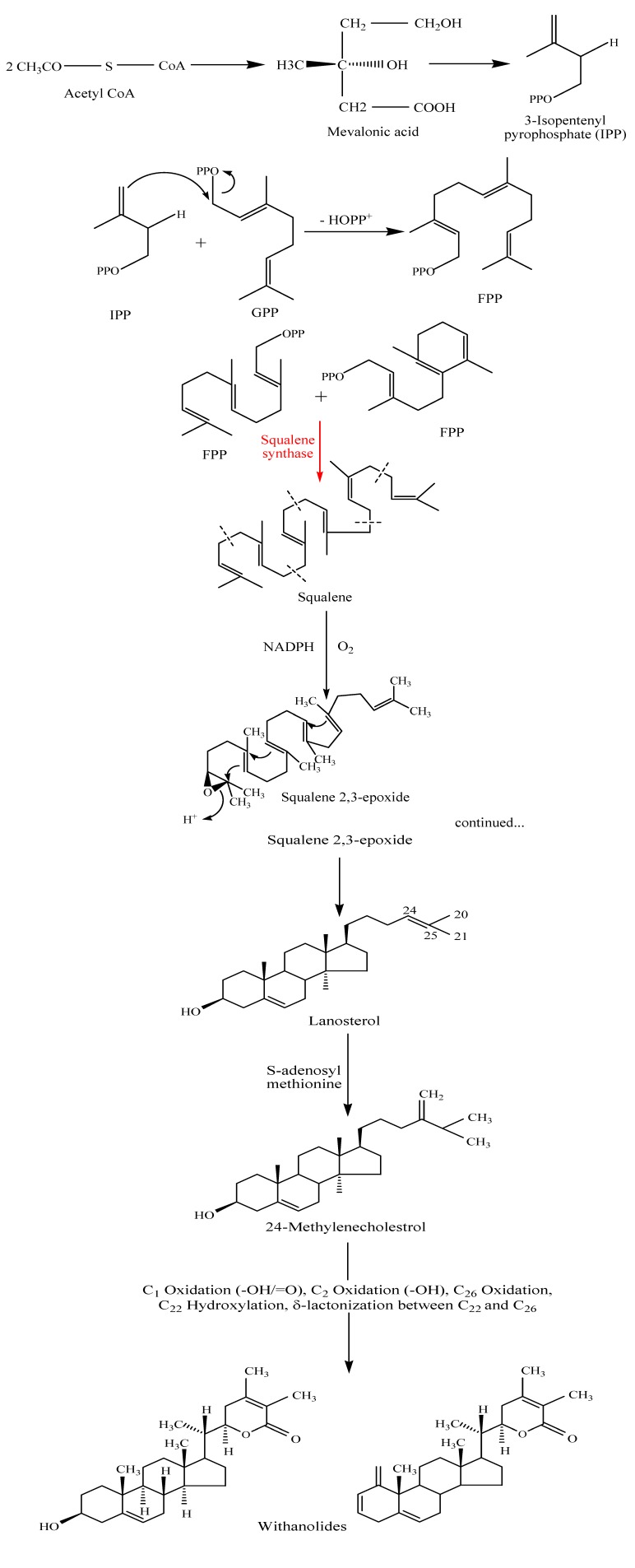
Overview of the most important steps in the withanolide biosynthetic pathway.

The biosynthetic pathways of withanolides and other chemical constituents of *W. somnifera* are not fully known, and there is very little information about their biogenetic aspects [38,39,40]. It has been reported that, except for a very few exceptions, the plants that synthesize the 20-H withanolides are unable to produce the 20-OH counterparts and vice versa [38]. Since withanolides are probably derived from cholesterol, this is a pertinent starting point to discuss their biosynthesis. The first step in the biosynthesis of cholesterol is the activation of acetate by its conversion to acetyl Co-enzyme A, abbreviated as acetylCoA. Two units of acetylCoA are combined and metabolized to mevalonic acid. Only the *R-* form of mevalonic acid is used by the living system to produce terpenes, while the *S-* form is metabolically inert. The (*R*)-mevalonic acid is converted into isopentenyl pyrophosphate (IPP) through the loss of one carbon atom. The molecule of 3-isopentenyl pyrophosphate (IPP) can condense in a head-to-tail manner with its isomer, 3,3-dimethyl allyl pyrophosphate (DMAPP), to give geranyl pyrophosphate (GPP). A condensation reaction of *trans* geranyl pyrophosphate with another molecule of IPP yields farnesyl pyrophosphate (FPP). The enzyme squalene synthase catalyses the condensation of two molecules of farnesyl pyrophosphate in a head-to-head manner in the presence of NADPH to produce squalene. Oxidation of squalene by atmospheric oxygen is catalyzed by NADPH-linked oxide to afford squalene 2,3-epoxide. The latter undergoes ring closure to form lanosterol which is then converted into a variety of different steroidal triterpenoidal skeletons. The bioconversion of lanosterol to 24-methylenecholesterol is still not fully understood. The sequence of reactions and intermediates may also differ slightly among organisms. 24-Methylenecholestrol may be a biosynthetic precursor of steroidal lactones. It has been proposed that the hydroxylation in C_22_ and δ-lactonization between C_22_ and C_26_ of 24-methylenecholestrol yields withanolides (see Figure 2). It has also been suggested that the α,β-unsaturated ketone in ring A of common withanolides may be produced through the sequence 20-23 [41,42,43].

**Figure 3:**
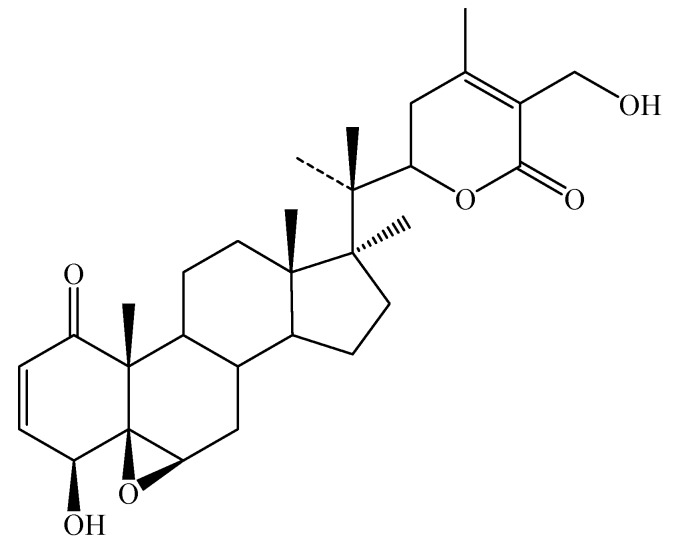
Structure of withaferin A.

Withaferin A (4β,27-dihydroxy-1-oxo-5β,6β-epoxywitha-2-24-dienolide, Figure 3) was the first member of this group of compounds to be isolated from the well-known South-Asian medicinal plant, *W. somnifera*. The structural novelty and interesting biological activities elicited by this compound led to a thorough chemical investigation of the plant and numerous compounds with similar structural features were isolated [28,30,36]. 

**Figure 4:**
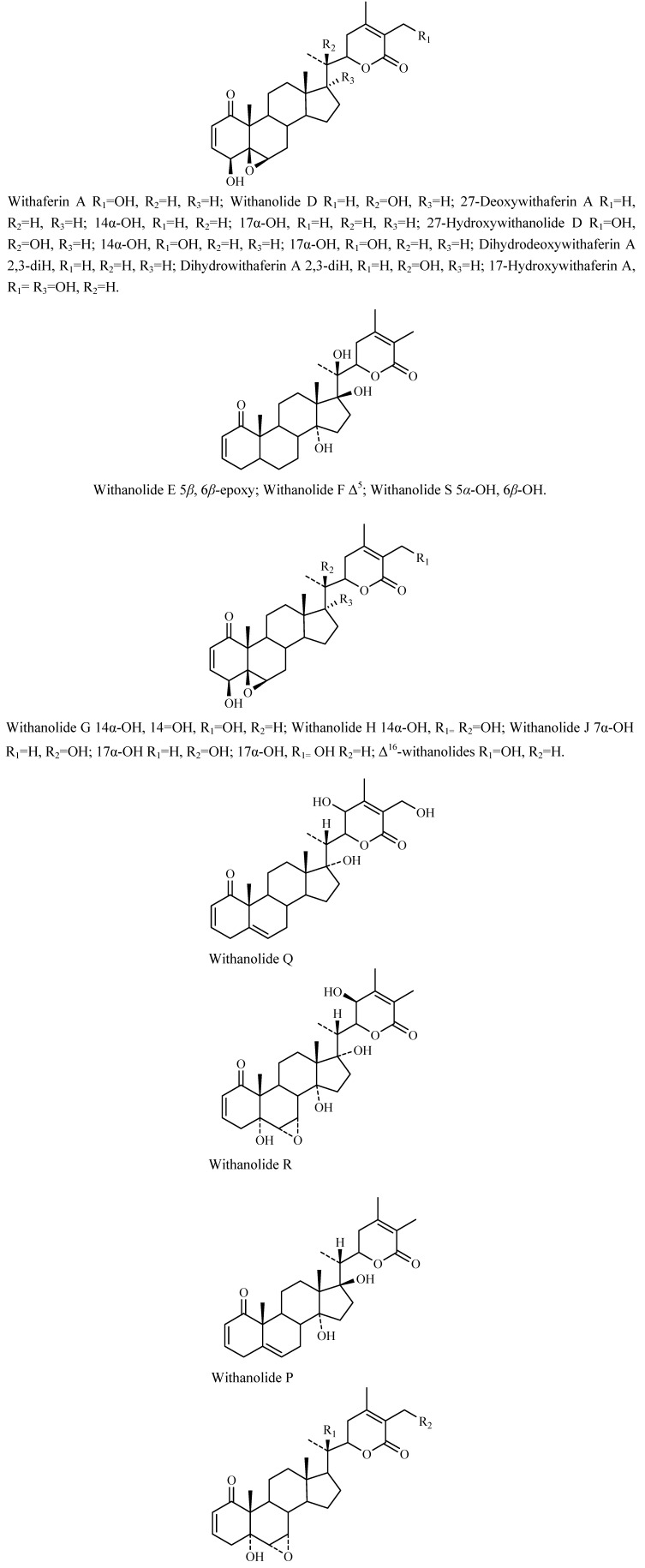
Different structures of withanolides.

Lavie’s group [44] elucidated the structure of withaferin A in leaves of this plant, which is mainly valued for its anti-cancerous properties. The yields of withaferin A from intact plants of *Withania* spp. (Israel Chemotype) are 0.2-0.3% of DW of leaves [45]. Gupta *et al*. [46] have performed a quantitative analysis of Indian chemotypes of *W. somnifera* by TLC densitometry and observed that withaferin A is totally absent in roots, stems, seeds and persistent calyx of fruits of intact plants but present in leaves (1.6%). Today over 130 withanolides from Solanaceae genera are known, mostly occurring in free form, but in a few cases also as glycosides [28], some of which are shown in Figure 4.

Withanone 17α-OH, R_1_=H, R_2_=H; Withanolide T 14α-OH, 17α-OH, R_1_=OH; Withanolide B R_1_=H, R_2_=H; Withanolide A R_1_=OH, R_2_=H.

## Other compounds

**Figure 5:**
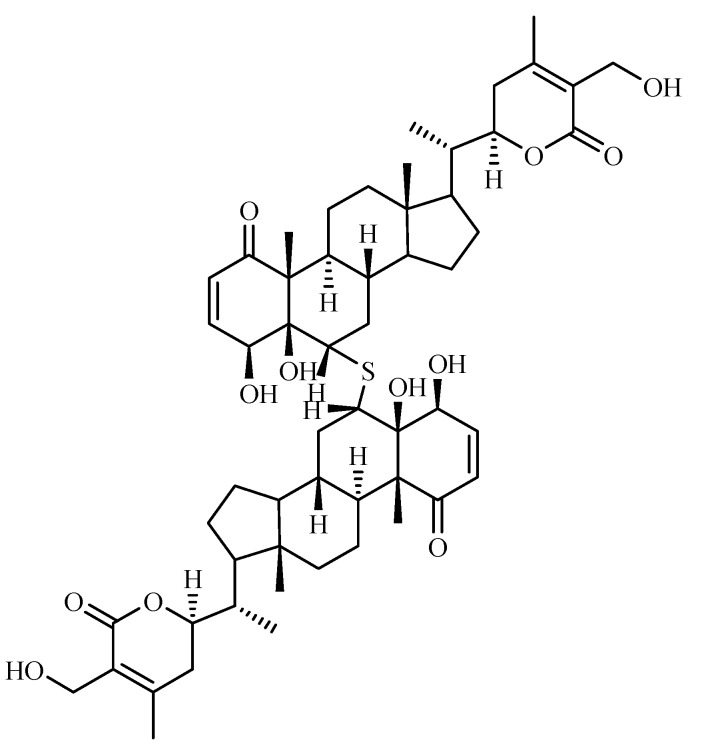
Ashwagandhanolide, a new compound isolated from *W. somnifera.*

**Figure 6:**
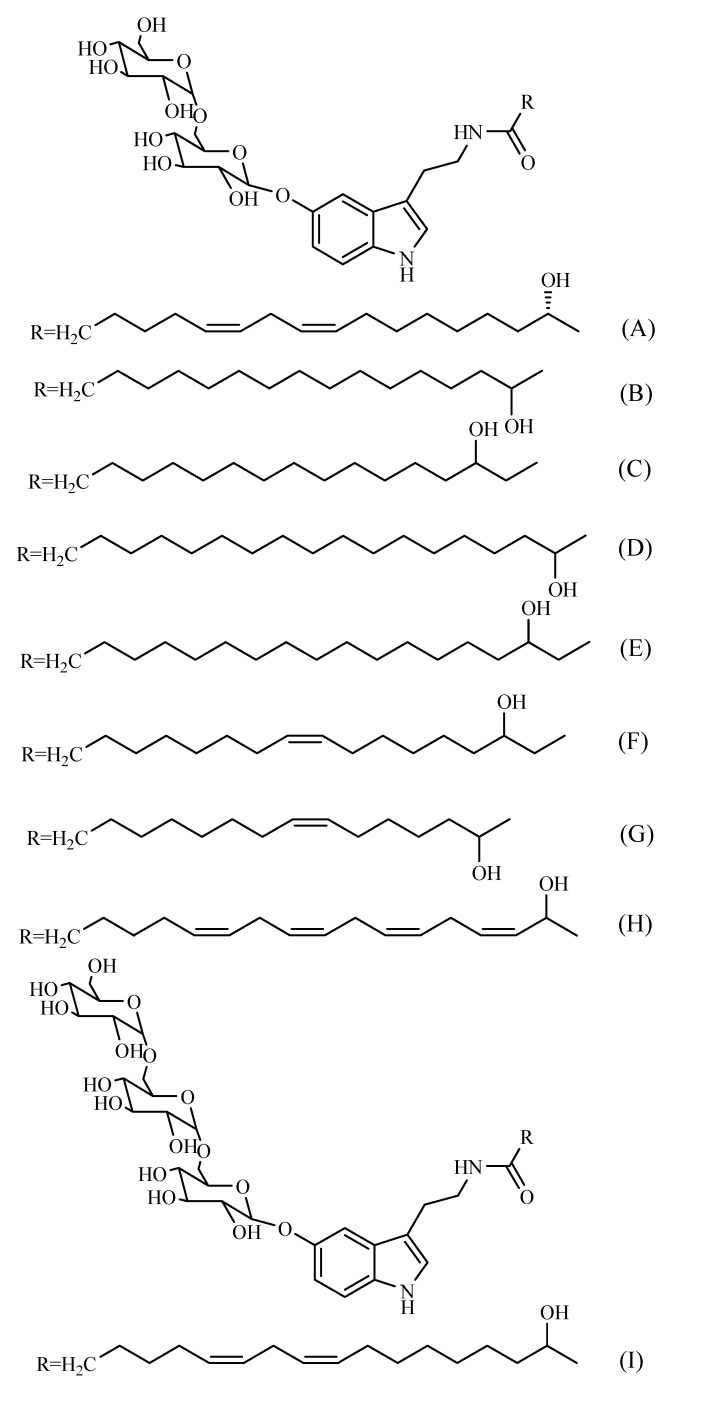
Different withanamides (A-I) isolated from *W. somnifera* fruits.

Examination of *W. somnifera* roots has resulted in the isolation of a new dimeric thiowithanolide, named ashwagandhanolide (Figure 5) [47]. A bioassay-guided purification of the methanolic extract of *W. somnifera* fruits yielded withanamides A-I (Figure 6). The structure of these compounds was determined by using serotonin, glucose and long-chain hydroxyl fatty acid moieties [48]. 

In their quantitative analysis of Indian chemotypes of *W. somnifera* by TLC densitometry, Gupta *et al*. [46] detected alkaloids in all the abovementioned plant parts, with the highest content found in leaves. This is in contrast to the general belief that tropane alkaloids are restricted to the roots of *Withania* spp. Extraction with 45% alcohol yields the highest percentage of alkaloids. The isolation of nicotine, somniferine, somniferinine, withanine, withananine, pseudowithanine, tropine, pseudotropine, 3α-tigloyloxytropane, choline, cuscohygrine, *dl*-isopelletierine and new alkaloids anaferine and anhygrine has been described [14,49]. The reported total alkaloid content in the roots of Indian *W. somnifera* varies between 0.13 and 0.31%, though much higher yields (up to 4.3%) have been recorded in plants of other regions/countries. In addition to the alkaloids, the roots are reported to contain starch, reducing sugars, hentriacontane, glycosides, dulcitol, withanicil, an acid and a neutral compound. The leaves are reported to contain five unidentified alkaloids (yield 0.09%), chlorogenic acid, calystegines (nitrogen-containing polyhydroxylated heterocyclic compounds) withanone, condensed tannin and flavonoids. The berries have amino acids. Four types of peroxidases have been purified and characterized from *W. somnifera* roots [14,50].

## Pharmacological activities of withanolides

The pharmacological activity of *W. somnifera* extracts has been summarized recently by Gupta and Rana [49]. Historically, *W. somnifera* has been used as an antioxidant, adaptogen, aphrodisiac, liver tonic, antiinflamatory agent and astringent and more recently as an antibacterial, antihyperplycemic and antitumoral, as well as to treat ulcers and senile dementia. 

The active principles of *W. somnifera* have been tested for antioxidant activity by observing the levels of the major free-radical scavenging enzymes, superoxide dismutase, catalase and glutathione peroxidase, in the rat brain frontal cortex and striatum. The increase in these enzymes after treatment with withanolides represent enhanced antioxidant activity and a corresponding protective effect on neuronal tissue, suggesting that the antioxidant effect of *W. somnifera* in the brain may be responsible for its diverse pharmacological properties [51]. Similarly, oral administration of *W. somnifera* extracts prevented an increase in lipid peroxidation in mice and rabbits [52]. 

Anxiolitic and antidepressant actions of the bioactive withanolides have been assessed in rats [53]. Withanolides reduced rat brain levels of tribulin (an endocoid marker of anxiety) when the levels of this compound were increased by administration of pentylenetetrazole, an anxiogenic agent. The antidepressant effect of withanolides is comparable with that induced by imipramine in the forced swim-induced “behavioural despair” and “learned helpessness” test. In a rat model withanolides were able to decrease the number and severity of chronic stress-induced ulcers, reverse chronic stress-induced inhibition of male sexual behavior, chronic stress-induced immunosuppression and also increased peritoneal macrophage activity [54]. It has also been demonstrated that methanolic extracts of *Withania* reduce ulcer index, volume of gastric secretion, free acidity, and total acidity [55]. 

The effects of sitoindosides VII-X and withaferin isolated from aqueous methanol extracts of *W. somnifera* roots were studied on brain cholinergic, glutamatergic and GABAergic receptors in rats. The data suggest the bioactive compounds preferentially influence events in the cortical and basal forebrain cholinergic-signal transduction cascade. The cognition-enhancing and memory-improving effects of *W. somnifera* extracts can be partly explained by the drug-induced enhancement of cortical muscarinic acetylcholine receptor capacity [56]. In general, Ashawagandha has been used traditionally and commonly as a tonic and nootropic agent. It has also been associated with improvements in scopolamine-induced memory deficits in mice [57]. Methanolic extracts of the plant have been reported to induce neurite extension [58] and to contain withanolides such as withanolide A, withanoside IV and withanoside VI, which induce neurite outgrowth in human neuroblastome SH-SY5Y [59]. In Aβ(25-35)-induced damaged cortical neurons, withanolide A, withanoside IV and withanoside VI showed neuritic regeneration and synaptic reconstruction. Dentritic atrophy was completely prevented by treatment with these withanolides, particularly withanoside IV and VI [60]. 

Neuroleptic-induced catalepsy has been used as an animal model for screening drugs for Parkinson’s disease. Hope of treatment for this disease has been offered by the inhibitory effects of *W. somnifera* extracts on haloperidol or reserpine-induced catalepsy in mice [61]. The antiparkinsonian effect of *W. somnifera* extracts has also been attributed to potent antioxidant, antiperoxidative and free radical quenching properties [62]. 

The extracts of *W. somnifera* have shown antiinflamatory effects in a variety of rheumatological conditions, reducing, for example, Freund’s complete adjuvant-induced inflammation in rats and decreasing to undetectable levels the α2-glycoprotein found only in inflamed rat serum [63]. In another study, *W. somnifera* caused suppression of a2-macroglobulin, an indicator for antiinflamatory drugs in rat serum inflamed by injection of carrageenan suspension [64]. The extracts also caused a significant reduction in both paw swelling and bony degenerative changes in Freund’s adjuvant-induced arthritis as observed by radiological examination [65]. Rats injected with formaline in the hind leg footpad showed decreased absorption of ^14^C-glucose in rat jejunum, glucose absorption being maintained at the normal level by *Withania* extracts, which produced an antiinflamatory effect [66]. 

The traditional antihyperglycemic and antidyslipidemic activities of *W. coagulans*, popularly known as Indian cheese marker, have been recently confirmed [67]. Withanolides isolated from *W. coagulans* fruits show significant inhibition of the postprandial rise in hyperglycemia post-sucrose load in normoglycemic rats as well as streptozocin-induced diabetic rats.

*W. somnifera* extracts have a chemopreventive effect on skin cancer in mice induced by 7,12-dimethylbenz[a]antracene. This activity is thought to be partly due to the antioxidant/free radical scavenging activity of the extract [68]. Recently, bioactive properties of withaferin A have been reported: cytoskeletal architecture alteration by covalently binding annexin II [69], antitumor capacity by inhibition of proteasomal chymotrypsin-like activity [70], and apoptosis induction through the inhibition of protein kinase C [71]. In relation to the apoptosis-inducing mechanism of withaferin A, Oh *et al*. [72] have demonstrated that it is associated with the activation of caspase-3 and the translocation of cytochrome *c* from the mitochondria to the cytosol, as well as the cleavage of PLC-γ1 (a substrate protein of caspases), whereas ectopic expression of Bcl-2 oncoprotein significantly attenuates withaferin A-induced apoptosis. 

*Withania* alkaloids have a prolonged hypotensive, bradycardic and respiratory-stimulant action in dogs [73]. It has been found that the hypotensive effect is mainly due to autonomic ganglion blocking action as well as a depressant action on the higher cerebral centers. The alkaloid stimulates the vasomotor and respiratory centers in the brain stem of the dogs. The cardio-inhibitory action appeared to be due to ganglion blocking and direct cardio-depressant actions. 

## A biotechnological approach to withanolide production

Many biotechnological studies of *Withania* species, especially *W. somnifera*, have been carried out to enhance the production of their active compounds. Large-scale plant cell cultures may be cost-effective and also allow the production of higher amounts of withanolides in a relatively short period of time. 

Tissue cultures of an Indian chemotype of *W. somnifera* from axillary meristems using MS agar medium supplemented with 2,4-D, IAA, NAA, BA, coconut milk or kinetin, either alone or in combination, have been reported [74]. Calli were formed on medium containing 2.0 mg 2,4-D/L and 0.2 mg Kin/L. Suspension cultures were initiated from callus tissue in liquid MS medium supplemented with 2.0 mg 2,4-D/L, 0.2 mg Kin/L and 2.0 mg BA/L. Multiple shoot cultures were also grown in MS agar medium containing 2.0 mg BA/L. Callus cultures failed to synthesize withanolides, but multiple shoot cultures synthesized significant amounts; their concentrations were highest with 2.0 mg BA/L and 10% coconut milk. Multiple shoot cultures of *W. somnifera* from single shoot tip explants and their potential for the production of withaferin A and withanolide D have also been investigated [75]. Shoot tips grown on MS medium supplemented with BA (1 mg/l) induced 10.0 microshoots per explant and shoot cultures accumulated withaferin A (0.04 %) and withanolide D (0.06%). Supplementation of MS solid agar medium with 1.0 mg BA/L and 4% sucrose enhanced accumulation of both withaferin A (0.16%) and withanolide D (0.08 %). MS liquid medium containing 1.0 mg BA/L and 10% coconut milk favoured a maximum increase in biomass (27 fold), induced microshoots (37.6) as well as accumulation of withaferin A (0.14%).

Direct rooting from leaf explants of *W. somnifera* has been achieved on half-strength MS medium supplemented with 15 g/l sucrose, and different concentrations of growth regulators [76]. The roots were cultured on MS liquid medium for the establishment of root-organ cultures with the same plant growth regulators and incubated on an orbital shaker at 80 rpm at 25±2 ^◦^C. The concentration of alkaloids increased compared to field grown roots. The maximum concentration of withanolides (10 mg/g dry weight) was obtained in the bioreactor. Recently, withanolide A biogeneration in shoot cultures of *W. somnifera* has been reported [77]. Multiple shoot cultures of two experimental lines of *W. somnifera* were established using nodal segments as explants. The hormonal combinations of benzyl adenine and kinetin influenced morphogenetic response as well as differentially modulating the level of biogeneration of withanolide A in the *in vitro* shoots of the two lines. The production of withanolide A in the cultures varied considerably (*ca.* 10-fold, 0.014 to 0.14 mg/g fresh weight) according to the hormone composition of the culture media as well as the genotype used as the explant source. The shoot culture of experimental lines cultivated at 1.0 ppm of BAP and 0.5 ppm of kinetin displayed the highest concentration of withanolide A in the green shoots of 0.238 %. 

## Hairy root cultures as a source of withanolides

In the two last decades, the hairy root system based on *Agrobacterium rhizogenes* inoculation has become popular as a method of producing secondary metabolites synthesized in plant roots [78,79]. Unorganized plant tissue cultures are frequently unable to produce secondary metabolites at the same levels as the intact plant. The hairy root phenotype is characterized by fast, hormone-independent growth, lack of geotropism, lateral branching and genetic stability. The secondary metabolites produced by hairy roots arising from the infection of plant material by *A. rhizogenes* are the same as those usually synthesized in intact parent roots, with similar or higher yields [80]. This feature, together with genetic stability and generally rapid growth in simple media without phytohormones, makes them especially suitable for biochemical studies not easily undertaken with root cultures of an intact plant. Banerjee *et al*. [81] carried out hairy root transformation of *W. somnifera* by three different strains of *A. rhizogenes* (A4, LBA 9402 and LBA 9360) and analyzed the specificity and frequency of their withanolide production with special reference to withaferin A. The best response in terms of transformation ability and growth of the hairy roots was obtained with strain A4, followed by LBA 9402; LBA 9360 failed to induce a transformation event. The production of withaferin A was studied in the A4-induced hairy root lines at different growth phases (4, 10 and 24 weeks) using HPLC (high performance liquid chromatography) and maximum levels were observed in the media and hairy roots of 10-week-old cultures. During the infection process *A. rhizogenes* transfers a part of the DNA (transferred DNA, T-DNA) located in the root-inducing plasmid Ri to plant cells and the genes contained in this segment are expressed in the same way as the endogenous genes of the plant cells [82]. Some *A. rhizogenes*, such as strain A4, have the T-DNA divided in two sections, the TR-DNA and TL-DNA, each of which can be incorporated separately into the plant genome. Two sets of pRi genes are involved in the root induction process: the *aux* genes located in the TR region of the pRi T-DNA and the *rol* (root loci) genes of the TL region [83]. The *ags* genes responsible for opine biosynthesis in the transformed tissues are also located in the TR region [84]. Opines are synthesized by plant transformed cells and are only used by *Agrobacterium* as a source of nitrogen and carbon. Due to the similarities between the *A. rhizogenes* and *A. tumefaciens* infection processes, and because both microorganisms are very closely related, it has been suggested that the most important *A. rhizogenes* oncogenes encode proteins involved in the regulation of plant hormone metabolism. Transformation of *W. somnifera* with wild type nopaline and octopine strains of *A. tumefaciens* has been reported [75]. The oncogenic strains showed different levels of virulence in the two genotypes studied, the main difference being found in the nature of the galls formed and in their subsequent morphological competence. Ten percent of the galls obtained after infection with nopaline strain N2:73 spontaneously developed shooty teratomas of altered phenotype. The shooty teratomas grew in unsupplemented basal medium and were able to synthesize both major withanolides of the parent plants. Withanolide synthesis in shooty teratomas was much higher (0.07–0.1% withaferin A and 0.085–0.025% withanolide D) than in non-transformed shoot cultures. 

*Aux* genes provide transformed cells with an additional source of auxin [85,86], but they do not seem essential for developing hairy root disease [79]. However, *rol* genes have functions that are most likely other than that of producing mere alterations in plant hormone concentrations [87]. Several authors have investigated the effect of TR and TL regions of *A. rhizogenes* on the growth and morphology of transformed roots and plants, but until now there have been few studies on the direct effects of oncogenes on secondary metabolism. As previously reported, a correlation exists between the expression of the *rol*C gene and the production of tropane alkaloids [88,89,90], *Catharanthus roseus* alkaloids [91], and ginsenosides [92]. No correlation between *rol*A and *rol*B expression and secondary metabolism was found in any of these studies. Moyano *et al*. [93] showed that the inoculation of leaf sections of tobacco, *Duboisia* hybrid and *Datura* metel plants with the A4 strain of *A. rhizogenes* induced transformed roots with the capacity to produce putrescine-derived alkaloids such as nicotine, hyoscyamine and scopolamine. The obtained hairy roots generally presented two morphologies: typical hairy roots with a high alkaloid-producing capacity, and callus-like roots with faster growth and lower alkaloid production. The *aux*1 gene of *A. rhizogenes* located in the TR-DNA of *A. rhizogenes* was detected in all roots showing callus-like morphology, but only in 25-60% of the established root cultures showing typical hairy morphology. These results demonstrate a significant role of *aux* genes in the morphology of transformed roots and the importance of typical hairy root morphology in the production of scopolamine. The studies with *Panax ginseng* hairy roots also support the effects of the genes located in the TR-DNA on root morphology and secondary metabolism [94]. Bandyopadhyay *et al*. [25] have reported the presence of TR-DNA in all the transformed callus lines of *W. somnifera* obtained after infection with *A. rhizogenes* A4, thus confirming the effects of *aux* genes on root line phenotypes. Recently, Mirjalili *et al*. [95] showed that the inoculation of leaf sections of *W. coagulans* with *A. tumefaciens* strain C58C1 (pRiA4) induced transformed roots with two morphologies: callus-like roots (CR) with a high capacity to produce withanolides and typical hairy roots (HR) with faster growth capacity and lower withanolide accumulation. The presence of the *aux*1 gene has been confirmed in 100% of the root lines displaying callus-like morphology, but in only 12.5% of roots with typical hairy root morphology.

Withanolide composition and *in vitro* culture of an Italian chemotype of *W. somnifera* have been reported [96]. Withanolide production by *in vitro* cultures (callus, shoots, leaves and roots transformed by *A. rhizogenes*, derived from the Sicilian plant) was investigated on MS media either supplemented with BA or 2,4-D, or without any plant growth regulators. Limited production was observed in shoot and callus cultures and no withanolides were detected in hairy roots. Withanolide production by hairy root cultures of *W. somnifera* transformed with *A. rhizogenes* (LBA 9402) has also been reported [97]. Hairy roots grew on MS medium containing 3% (w/v) sucrose in the absence of exogenous plant growth regulators. Root cultures synthesized several withanolides, from which withanolide D was isolated and identified. The productivity of withanolide D in transformed roots (0.181 mg/L) was higher than in untransformed root cultures (daily production of 0.181 and 0.026 mg/L, respectively). 

Developmental variability and withanolide production were introduced into *W. somnifera* using genetic transformation by different strains of *A. rhizogenes*. After inoculation, typical transformed root lines, transformed callus lines, and rooty callus lines were produced and their growth rates and withasteroid accumulation were studied. Accumulation of withaferin A was maximum (0.44% dry weight) in the transformed hairy root lines. All the rooty callus lines accumulated both withaferin A and withanolide D. Some of the callus lines produced both withaferin A (0.15–0.21% dry weight) and withanolide D (0.08–0.11% dry weight), and they grew faster than the transformed root lines [25]. 

## Conclusions

By applying novel study concepts and objective scientific analysis, drugs used in traditional medicine may be a rich source of new medicines to treat intractable diseases. In this context, Aswagandha, chemically rich with its varied content of active compounds, such as withanolidess, sitoindosides and many useful alkaloids, and used for centuries to treat a wide range of diseases, constitutes a promising candidate as a multi-purpose medicinal agent. However, more clinical trials need to be carried out to support its therapeutic use. For example, although the high potential of withanolide A for neuronal regeneration is well-known, it would be dangerous to simply imply that this compound could be an excellent anti-dementia drug. It would first be necessary to investigate the side effects of the bioactive compounds and their possible interactions, and to develop more clinical experiments. 

Nevertheless, *Withania* active components have promising activities, and biotechnological production could offer an alternative to conventional cultivation. Several laboratories have recently developed plant cell and hairy root cultures for the production of the most important bioactive components of *Withania* extracts, withaferin A and withanolide A. Although withanolide production by *in vitro* cultures is still far from the levels required for economical exploitation, these studies are useful tools to obtain greater understanding of the withanolide metabolic pathway, allowing the application of plant metabolic engineering techniques to improve the biotechnological production of *Withania* bioactive compounds. 
